# The Association of Antarctic Krill *Euphausia superba* with the Under-Ice Habitat

**DOI:** 10.1371/journal.pone.0031775

**Published:** 2012-02-23

**Authors:** Hauke Flores, Jan Andries van Franeker, Volker Siegel, Matilda Haraldsson, Volker Strass, Erik Hubert Meesters, Ulrich Bathmann, Willem Jan Wolff

**Affiliations:** 1 Institute for Marine Resources and Ecosystem Studies (IMARES), Texel, The Netherlands; 2 Johann Heinrich von Thünen Institute for Sea Fisheries, Hamburg, Germany; 3 Department of Marine Ecology, University of Gothenburg, Fiskebäckskil, Sweden; 4 Alfred Wegener Institute for Polar and Marine Research (AWI), Bremerhaven, Germany; 5 Center for Ecological and Evolutionary Studies, University of Groningen, Haren, The Netherlands; National Institute of Water & Atmospheric Research, New Zealand

## Abstract

The association of Antarctic krill *Euphausia superba* with the under-ice habitat was investigated in the Lazarev Sea (Southern Ocean) during austral summer, autumn and winter. Data were obtained using novel Surface and Under Ice Trawls (SUIT), which sampled the 0–2 m surface layer both under sea ice and in open water. Average surface layer densities ranged between 0.8 individuals m^−2^ in summer and autumn, and 2.7 individuals m^−2^ in winter. In summer, under-ice densities of Antarctic krill were significantly higher than in open waters. In autumn, the opposite pattern was observed. Under winter sea ice, densities were often low, but repeatedly far exceeded summer and autumn maxima. Statistical models showed that during summer high densities of Antarctic krill in the 0–2 m layer were associated with high ice coverage and shallow mixed layer depths, among other factors. In autumn and winter, density was related to hydrographical parameters. Average under-ice densities from the 0–2 m layer were higher than corresponding values from the 0–200 m layer collected with Rectangular Midwater Trawls (RMT) in summer. In winter, under-ice densities far surpassed maximum 0–200 m densities on several occasions. This indicates that the importance of the ice-water interface layer may be under-estimated by the pelagic nets and sonars commonly used to estimate the population size of Antarctic krill for management purposes, due to their limited ability to sample this habitat. Our results provide evidence for an almost year-round association of Antarctic krill with the under-ice habitat, hundreds of kilometres into the ice-covered area of the Lazarev Sea. Local concentrations of postlarval Antarctic krill under winter sea ice suggest that sea ice biota are important for their winter survival. These findings emphasise the susceptibility of an ecological key species to changing sea ice habitats, suggesting potential ramifications on Antarctic ecosystems induced by climate change.

## Introduction

Antarctic krill *Euphausia superba* often dominates the zooplankton community in numbers and biomass south of the Antarctic Polar Front (APF). It is a globally important fisheries resource [1], [2], [3], with recent estimates of the total stock biomass ranging between 169 and 379 million metric tons [4], [5]. Antarctic krill has adapted to almost the entire range of marine habitats in the Southern Ocean, including the abyssal plains [6] and the underside of pack-ice [7], [8]. Its potential distribution covers large parts of the Southern Ocean, with the exception of the inner shelf areas of the Ross and Weddell Seas [9], [10]. In high-abundance regions, e.g. near the Antarctic Peninsula, in the Scotia Sea and the Scotia Arc archipelagos, Antarctic krill is a highly influential factor in the ecosystem, capable of grazing as much as 55% of the net primary production [11]. It also is often a dominant prey item in the diet of many higher predators [12], [13], [14].

The life cycle and winter survival of Antarctic krill is closely linked with sea ice [15]. An analysis of historical abundance data from the entire Southern Ocean highlighted the significance of this association by showing that krill density in summer is positively correlated with the areal sea ice extent in the preceding winter [16]. Until recently, knowledge on krill aggregations at the underside of sea ice has been limited to semi-quantitative observations on small spatial scales provided by divers and submersible camera systems [8], [17], [18], [19]. Under-ice data collected during summer with upward-looking sonars provided first evidence of elevated krill concentrations under pack-ice, in a narrow band several kilometres away from the ice margin [20]. Yet the extent to which Antarctic euphausiids and their larvae use the ice-water interface layer as a habitat is poorly investigated, because most quantitative sampling techniques integrate krill abundance over a depth range of at least 50 m from the surface [1], [3], [21], [22], [23], and the hydro-acoustic technology used to date has not been capable to resolve the upper few meters of the water column [20], [24], [25].

In winter, ice algae accessible from the underside of ice floes constitute an important resource for larval [26], [27], [28] as well as postlarval Antarctic krill [8], [29]. The floating sea ice ensures low but comparatively constant light exposure of algae, allowing photosynthetic production, while phytoplankton production in the water column is suppressed by low light availability due to deep vertical mixing [30]. Data on Antarctic krill from the areas covered by pack-ice, however, are scarce because of logistical constraints [31]. The vertical distribution of Antarctic krill during winter is still under debate due to a general scarcity of winter data from the sea ice zone. One widely accepted assumption is that diel vertical migration during winter ranges between 100 m depth at night and more than 300 m at day [4]. If Antarctic krill relied significantly on ice algae as a food source in winter, however, a constant distribution at greater depth would be unlikely. To which extent Antarctic krill depends on sea ice to survive the dark months is therefore still unclear, and various alternative hypotheses, e.g. usage of lipid deposits, reduced metabolism, benthic feeding and starvation combined with shrinkage are being discussed [32], [33], [34], [35].

In order to investigate the importance of the ice-water interface layer for euphausiids and other macrofauna, a new sampling device was developed for the quantitative sampling of this environment, the Surface and Under Ice Trawl (SUIT [36]; see supporting information S1). Three expeditions conducted in the same area in the Lazarev Sea provided the opportunity to investigate seasonal and spatial patterns in the occurrence of euphausiids in the surface layer.

This study aims to

1. Quantify the density and the population structure of Antarctic krill in the immediate (0–2 m) surface layer, both under sea ice and in open water in summer, autumn and winter;
2. relate these data to environmental parameters, such as sea ice properties, light regime and hydrography;
3. assess the importance of the ice-water interface layer in relation to conventional standardised sampling of the 0–200 m depth layer.

## Materials and Methods

### 1. Ethics statement

All necessary permits were obtained for the described field studies. Permits as required by the Dutch *Wet bescherming Antarctica* (WBA) were issued by the Netherlands Ministry of Agriculture, Nature and Food Quality (WBA ANT/03/003, WBA ANT/05/004, WBA ANT/07/001).

### 2. Data collection

#### Research area

**Figure 1:**
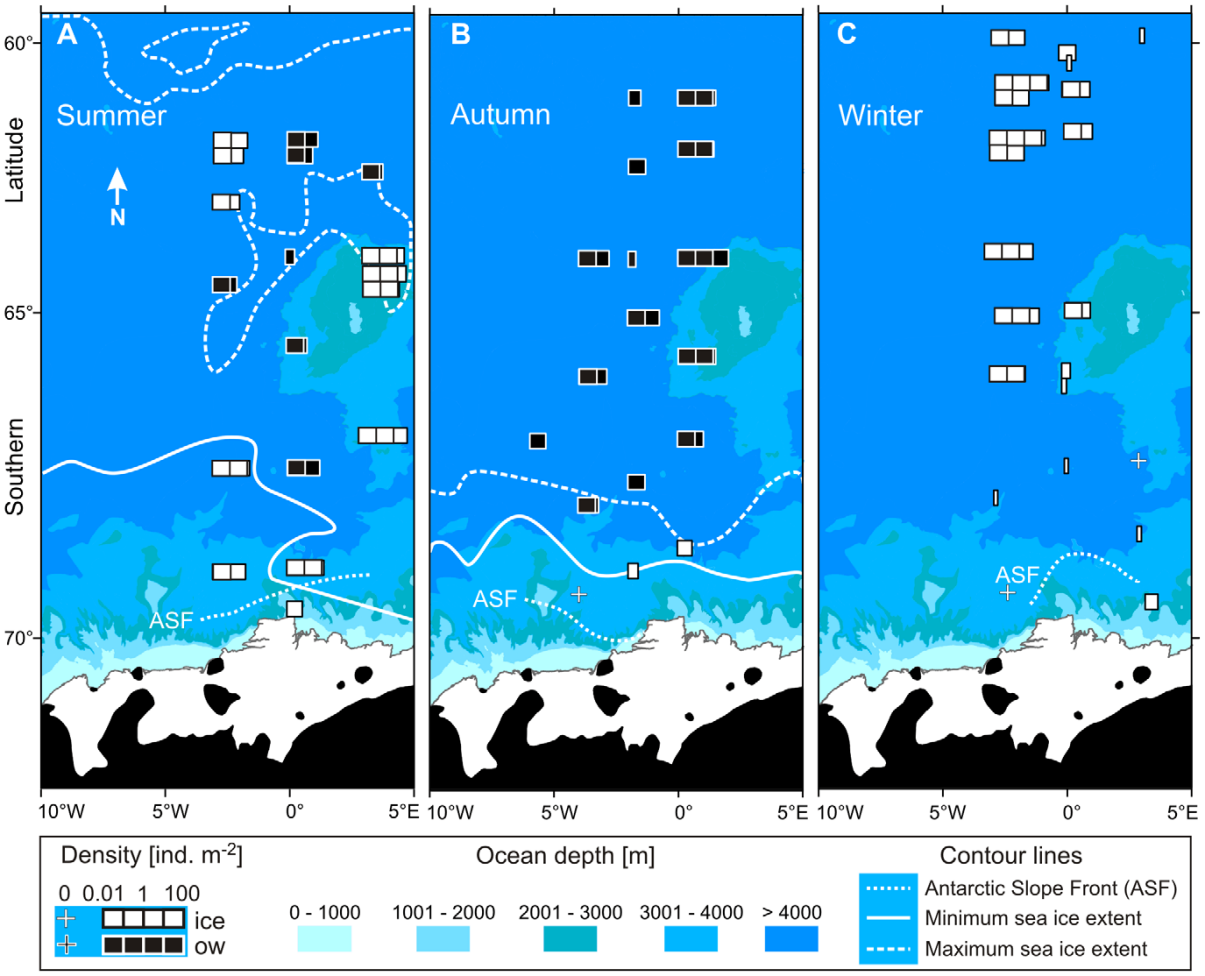
*Euphausia superba*. Spatial distribution of the surface layer density of postlarval krill in (**A**) summer (2007/2008), (**B**) autumn (2004), and (**C**) winter (2006). Minimum (Ice min) and maximum (Ice max) pack-ice extent during the sampling period is indicated by approximate 15% ice coverage derived from satellite data. The entire survey area was covered by sea ice in winter 2006. ASF: Antarctic Slope Front; ice = under-ice, ow = open water SUIT hauls.

Sampling was performed during three research missions of RV “*Polarstern*” in the Lazarev Sea (Southern Ocean) in austral summer (ANT XXIV-2, November 28^th^ 2007 to February 4^th^ 2008), autumn (ANT XXI-4, March 27^th^ to May 6^th^ 2004), and winter (ANT XXIII-6, June 17^th^ to August 21^st^ 2006). The expeditions were part of a multi-year field experiment embedded in the interdisciplinary LAzarev Sea KRIll Study (LAKRIS). The surveys sampled a regular station grid covering the seasonal sea ice zone, from 6°W to 3°E and from 60°S to the continental ice shelf at approximately 70°S (Figure 1). A detailed description of the area of investigation and the sampling scheme was provided in Flores et al. (2011) [37].

#### Surface layer sampling

Surface and Under Ice Trawls (SUIT [36]; see supporting information S1) were used to sample macrozooplankton and micronekton in the upper two metres of the water column. The net systems consisted of a steel frame with an approximately 2×2 m net opening with a 15 m long, 7 mm half-mesh commercial shrimp net attached (supporting information S1). In autumn 2004, a circular plankton net (diameter 50 cm, 0.3 mm mesh) was mounted inside the shrimp net to sample mesozooplankton and krill larvae. In summer (2007/2008) and winter (2006), the rear three meters of the net were lined with 0.3 mm plankton gauze. An Acoustic Doppler Current Profiler (ADCP, type Nortek EasyV) was used in summer and winter to estimate the amount of water entering the net mouth and to analyse its flow properties. A detailed description of the SUIT and its fishing properties is provided in supporting information S1. To avoid bias incurred by diel patterns in the depth distribution of euphausiids, sampling was predominantly conducted at night during all three surveys. During summer and winter, however, altogether 6 locations were sampled both at day and at night , in order to obtain a coarse appraisal of diel changes in the density of Antarctic krill in the surface layer. Trawling was conducted both while the ship was breaking through the ice, and along edges of large ice floes. During ice edge trawling, the SUIT was towed under the ice at a distance of approximately 10–80 m from the ice edge. During each trawl, changes in ship speed, proportional ice coverage [%], ice thickness [cm] and irregularities were estimated visually by an observer on deck, in closest possible proximity to the net.

The catch was immediately sorted on board. After the collection of all macrofauna >0.5 cm from either the entire sample or a representative subsample, the mesozooplankton fraction was preserved on 4% hexamine-buffered formaldehyde-seawater solution. In autumn 2004, the mesozooplankton fraction was obtained from the separate plankton net. Animals >0.5 cm collected from this net were combined with the shrimp net catch in subsequent analyses. Euphausiids were separated by species. Displacement volume and number of individuals of each species were noted. Euphausiids for length-frequency analysis were fixed in formaldehyde solution for 48 to 96 hours before sex determination and length measurement. Antarctic krill were measured from the front edge of the eye to the tip of telson (the “*Discovery*” method). *Thysanoessa macrura* were measured from the tip of the rostrum to the tip of the telson. Euphausiid furcilia larvae from the mesozooplankton fraction were identified to species level and counted.

#### Midwater sampling

Standardized double-oblique hauls to a depth of 200 m were conducted with a rectangular midwater trawl (RMT [38]) on all LAKRIS grid stations during the three expeditions. The sampling device consisted of an RMT 1 (mesh size = 0.33 mm) mounted above an RMT 8 with net openings of 1 and 8 m^2^, respectively. The RMT 8 had a mesh size of 4.5 mm at the opening and a codend mesh size of 0.85 mm. A calibrated digital flow meter (Hydro-Bios Kiel model 438 110) mounted outside the net opening allowed the volume of water passing through the net to be estimated. The average trawling speed was 2.5 knots (1.3 m s^−1^).

Immediately after catch retrieval, euphausiids were removed from the RMT 8 sample. If the sample size was larger than 1 litre, a representative subsample was analysed. Euphausiids were stored in 4% formalin-seawater solution for length measurements and maturity stage analyses. RMT 1 samples were immediately preserved in a 4% formaldehyde-seawater solution. A detailed description of the RMT sampling procedure was provided by Hunt et al. (2011) [23].

#### Hydrography and environmental data

Vertical profiles of temperature, salinity and density were obtained by lowering a CTD (Conductivity, Temperature, Depth) probe to depths varying between 1,000 m and the sea floor. The CTD (type SBE 911*plus*) *was* supplemented by an altimeter (type Benthos PSA-916) to measure the distance to the sea floor, a transmissometer (type Wet Labs C-Star) to measure the attenuation of light, and a chlorophyll-sensitive fluorometer (type Dr. Haardt BackScat; only in winter 2006 and summer 2007/2008). The temperature-salinity profiles were used to calculate the mixed layer depth (MLD [m]) for each station [39]. Solar radiation [W m^−2^] was measured by the ship's meteorological system. Bottom depth [m] was estimated for each station position using modeled global bathymetry from a publicly available database [40], [41]. The proportion of the distance the SUIT was towed under sea ice was used to estimate the percentage of sea ice coverage for each SUIT haul. SUIT hauls with a proportional ice coverage >10% were considered under-ice hauls. The full procedure of environmental data collection was explained in Hunt et al. (2011) [23].

### 3. Data analysis

Of the 56 quantitative SUIT hauls, 18 were conducted in summer, 16 in autumn, and 22 in winter (Table 1). Data from RMT hauls conducted at these SUIT locations were also analysed for comparison of krill densities and size distributions from the 0–2 m layer with corresponding datasets from standardised 0–200 m sampling. Hauls conducted at daytime (surface radiation >10 W m^−2^) were excluded from the autumn and winter datasets because of the known diel vertical migration behaviour of Antarctic euphausiids in these seasons [4], [24]. The number of animals caught was standardised to the surface area sampled and expressed as the density of individuals per square metre [ind.m^−2^].

**Table 1:** *Euphausia superba*.

		Summer			Autumn			Winter
		ow	ice	total	ow	ice	total	total (ice)
	*n*	7	11	18	13	3	16	19
Postlarval krill	Average	0.11	1.22	0.79	1.0	0.01	0.82	2.70
	Geometric mean	**0.06**	**0.48**	0.21	**0.15**	**0.00**	0.08	0.04
	Min	<0.01	0.01	<0.01	0.01	0	0	0
	Max	0.21	6.33	6.33	9.60	0.01	9.60	23.11
Furcilia larvae	Average	0	0	0	<0.01	0.08	0.02	0.06
	Geometric mean	0	0	0	**<0.01**	**0.08**	<0.01	0.01
	Min	0	0	0	0	0.05	0	0
	Max	0	0	0	0.03	0.11	0.11	0.48

Density of postlarval krill and furcilia larvae [ind. m^−2^] at the open surface and under ice (0–2 m) in summer (2007/2008), autumn (2004) and winter (2006). Geometric mean values significantly different from each other (ANOVA *p*<0.05) were printed in bold. ice = under-ice SUIT hauls; ow = open water SUIT hauls; *n* = number of samples.

Initial data exploration showed that the distribution of animal densities was highly skewed. Densities were therefore log (x+0.001)-transformed to conform with the model assumptions of subsequent statistical analyses. Variance analysis (ANOVA) was conducted to assess the significance of the effect of season and the presence of sea ice during SUIT hauls, and the interaction of the two factors. Test results for ice-open water comparisons within each sampling season were confirmed with the non-parametric Mann-Whitney U-test, which is robust against non-normal distribution of the data. Overall densities of animals for different years and ice conditions were expressed both as averages (i.e. arithmetic mean) and geometric means (i.e. the exponent of the mean of the log(x+0.001)-transformed densities).

Non-linear relationships between environmental variables and densities of postlarval Antarctic krill were investigated with Generalized Additive Models (GAM, [42]). The following environmental variables were included in the analysis:

- MLD;
- water temperature (0 m – MLD);
- water temperature (0–200 m);
- salinity (0 m – MLD);
- salinity (0–200 m);
- attenuation (0 m – MLD);
- attenuation (0–200 m); - potential temperature at depth of temperature maximum;
- chlorophyll a conc. (0–200 m; only 2006 and 2007/2008);
- ocean depth;
- solar radiation;
- proportional ice coverage during SUIT hauls;
- average ice thickness during SUIT hauls.

Due to multiple interactions of environmental variables with sampling season, separate models were computed for each season. Collinearity of variables was assessed by calculating correlation coefficients and variance inflation factors (VIF: e.g. [43]). VIF values above 10 are generally considered indicative of high collinearity [43]. Using a stepwise procedure, the variable with the highest VIF value was repeatedly removed until the VIF values of all remaining variables were below 10. Gaussian additive models were fitted using cubic splines and cross-validation to obtain the optimal degrees of freedom for each variable [44]. The optimal model was estimated by stepwise backward exclusion of insignificant model terms with the highest *P*-value, until the Akaike information criterion (AIC) reached a minimum. Sometimes the estimated degrees of freedom of smooth terms were so low that a linear relationship may have been sufficient. In that case the model was tested with parametric terms, and preferred if these were significant and the AIC was lower. Regression assumptions were assessed visually by plotting the response variable against fitted values and residuals against variables.

## Results

### 1. Hydrographical setting and ice coverage

The eastern Weddell Gyre fed Warm Deep Water of circumpolar origin into the survey area during all seasons. This water mass reached as far south as approximately 69°S, where the Antarctic Slope Front (ASF) was situated (Figure 1). Much colder and fresher waters of the Antarctic Coastal Current prevailed south of the ASF. The hydrography of the area was further influenced by current jets and eddies forming around the Maud Rise seamount [45]. In summer (2007/2008), sea ice extended north up to 60°S in December 2007, but retreated to a residual area south of 67°S in late January 2008 (Figure 1 A). In autumn (2004) the young pack-ice was largely confined to waters south of 68°S (Figure 1 B). Heavy pack-ice was present throughout the entire area of investigation in winter (2006) (Figure 1 C). The hydrography of the Lazarev Sea during the sampling of this study was described in detail by Hunt et al. (2011) [23].

### 2. Antarctic krill

#### Distribution and population structure

Antarctic krill was clearly the most abundant krill species and the only euphausiid caught in the 0–2 m surface layer in all three seasons. The average surface density was highest in winter (2006) (2.70 ind. m^−2^), followed by autumn (2004) (0.82 ind. m^−2^), and summer (2007/2008) (0.79 ind. m^−2^) (Table 1). In summer, densities were highest at the north and the south slopes of the Maud Rise seamount, between 64°S and 67°S 3°E (Figure 1 A). Elevated densities were concentrated north of 66°S along the 0° meridian in autumn (Figure 1 B), and at 3°W in winter (Figure 1 C). A few exceptionally high catches were obtained in these areas. These were one haul in summer (6.33 ind. m^−2^), one haul in autumn (9.60 ind. m^−2^), and two hauls in winter (17.86 and 23.11 ind. m^−2^).

**Figure 2:**
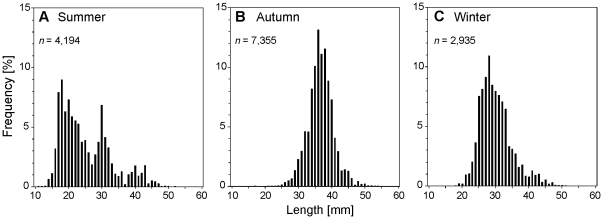
*Euphausia superba.* Length-frequency distributions of postlarval krill from the surface layer (0–2 m) on the LAKRIS grid in (**A**) summer (2007/2008), (**B**) autumn (2004), and (**C**) winter (2006).

The overall size range of postlarval Antarctic krill (13–54 mm) was largely similar in all three sampling seasons (Figure 2). Modes, however, occurred at different lengths in each season. In summer, the dominating fraction was juveniles, peaking at 18 mm. A second mode appeared at 30 mm (Figure 2 A). A single mode at 36 mm was observed in autumn (Figure 2 B). The mode in winter (28 mm) was considerably lower (Figure 2 C). No significant difference was apparent in any sampling season, when size distributions of postlarval Antarctic krill sampled by SUIT were compared with RMT length-frequency data (Table 2).

**Table 2:** *Euphausia superba*.

	Summer		Autumn		Winter	
	Surface	0–200 m	Surface	0–200 m	Surface	0–200 m
Mean length [mm]	25.2	26.7	36.7	35.2	30.1	33.7
Range [mm]	13–52	13–52	17–54	21–58	18–52	20–54
*p*	0.98		0.45		0.06	

Comparison of the size distributions of postlarval krill in the surface layer and the 0–200 m layer in summer (2007/2008), autumn (2004) and winter (2006). *p*: Kolmogorov-Smirnov test significance.

**Figure 3:**
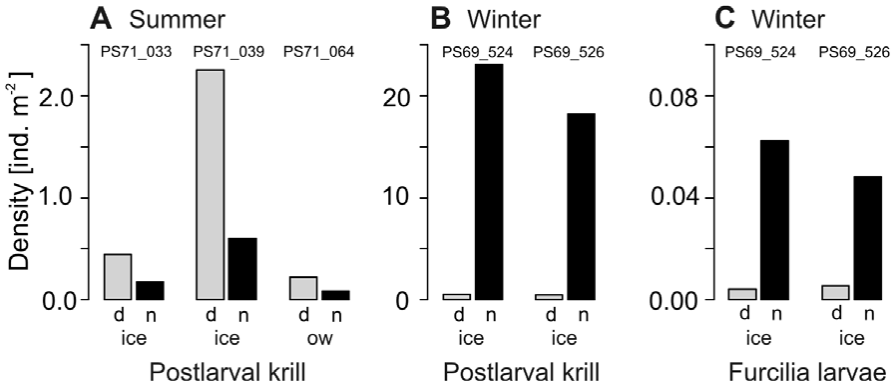
*Euphausia superba*. Day/night comparisons of the surface layer density of (**A**) postlarval krill at three locations in summer (2007/2008), (**B**) postlarval krill, and (**C**) furcilia larvae at two locations in winter (2006). Scaling of y-axis differs. Denotations above bars are location codes. d = daytime, night = night-time; ice = under-ice, ow = open water SUIT hauls.

A diel pattern in the surface density of postlarval Antarctic krill was apparent from the five day/night comparative locations that yielded a sufficient number of animals in summer and winter. In summer, densities of postlarval Antarctic krill were higher at day than at night, both under ice and in open water (Figure 3 A). In winter, a much more pronounced opposite pattern was recorded. The under-ice density at night was up to two orders of magnitude above daytime values (Figure 3 B).

Furcilia larvae of Antarctic krill were the dominant euphausiid larvae in the 0–2 m surface layer in autumn and winter, but had not yet developed from earlier stages in summer, because sampling was performed early in the season. In contrast to postlarval Antarctic krill, they were significantly more abundant under ice than in open water in autumn (Table 1). In winter, a diel pattern similar to postlarval krill was observed in the furcilia larvae (Figure 3 C).

#### Association with sea ice

The relation between the under-ice and open water 0–2 m surface layer densities of postlarval Antarctic krill significantly differed among sampling seasons (ANOVA: p<0.01). In summer, Antarctic krill density at the surface was significantly higher under ice than in open water. In autumn, the density of postlarval Antarctic krill was significantly lower under ice than in open waters. The integrated under-ice density, however, was based on only three of the 16 quantitative stations sampled on the LAKRIS grid in that season (Table 1). The highest local and average densities of postlarval Antarctic krill were recorded under the winter sea ice (Figure 1, Table 1).

**Figure 4:**
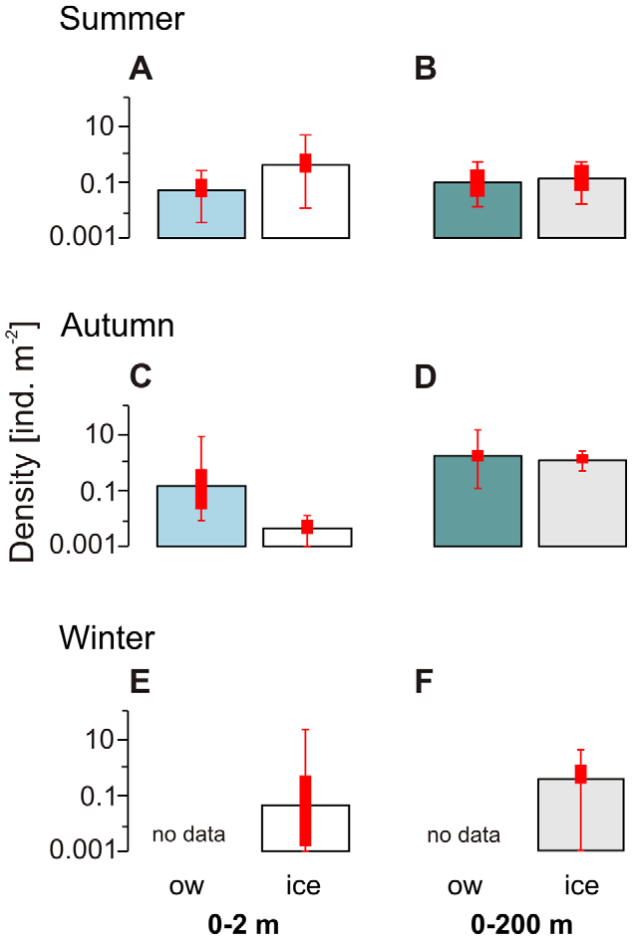
Under-ice versus open water comparison of geometric mean densities of postlarval *Euphausia superba* in (A–B) summer (2007/2008), (C–D) autumn (2004), and (E–F) winter (2006). (**A, C, E**) *Euphausia superba* from the 0–2 m layer, and (**B, D, F**) from the 0–200 m layer. Error bars denote value ranges. Bold red bars indicate 25% to 75% percentile ranges. ice = under-ice, ow = open water SUIT hauls.

The density of postlarval Antarctic krill in the 0–200 m stratum significantly differed among the sampling seasons (ANOVA: p<0.01), but was not significantly related to the presence of sea ice (ANOVA: p>0.1). In summer, both the average and geometric mean densities in the 0–200 m stratum were lower than in the 0–2 m layer in ice-covered waters, but above the values of the 0–2 m surface layer in open waters (Figure 4 A, B). In autumn, however, average and geometric mean densities were considerably higher in the 0–200 m depth layer than in the 0–2 m surface layer, both in open water and under ice (Figure 4 C, D). In winter, average krill density in the 0–200 m stratum was below the average density from the ice-water interface layer, mainly due to a few exceptionally high SUIT catches yielding densities up to 5 times above maximum values from the 0–200 m layer (Figure 1 C). Because of this high variability in the SUIT data, geometric mean densities showed the opposite pattern (Figure 4 E, F).

#### Relationship with environmental parameters

**Figure 5:**
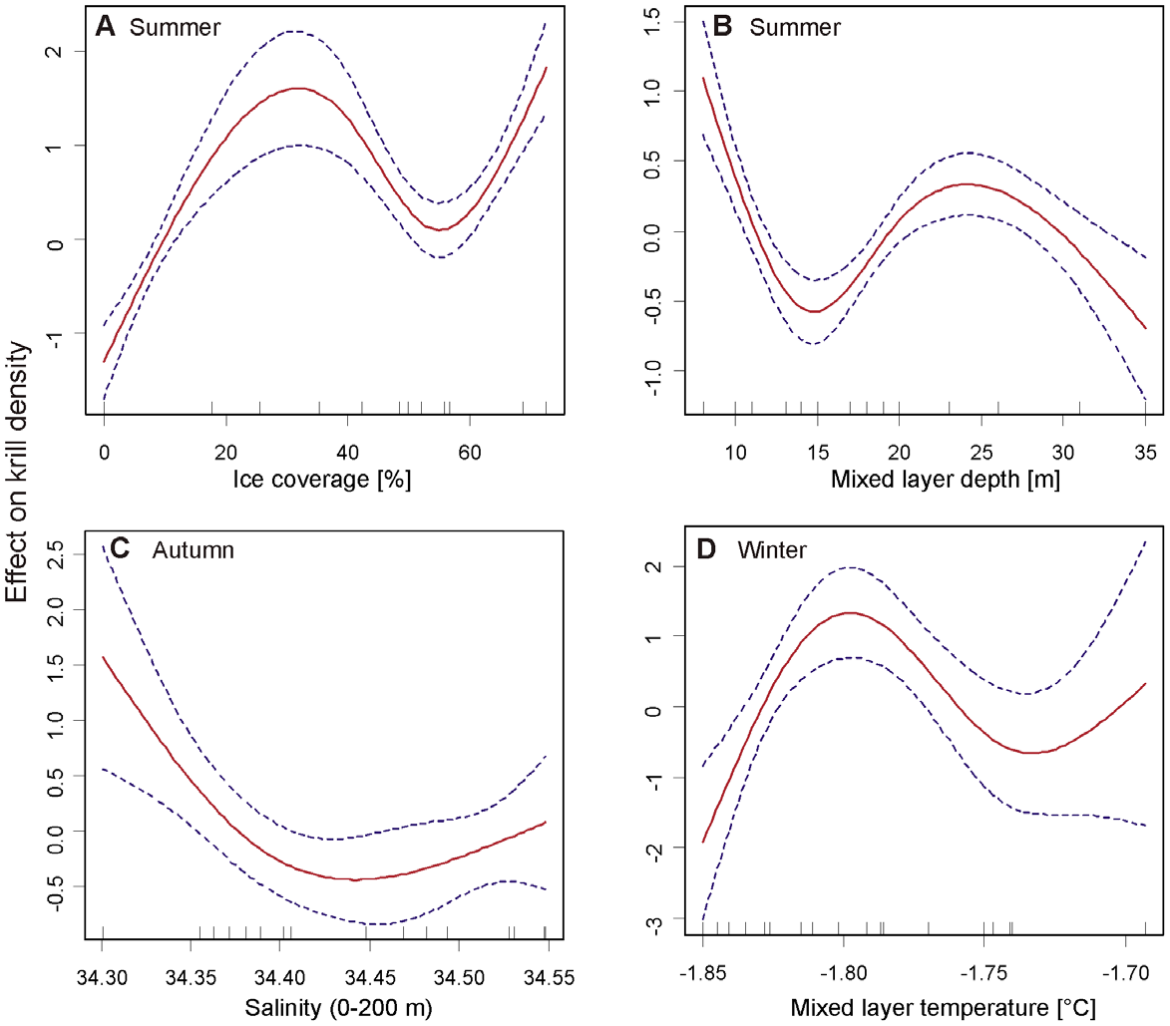
*Euphausia superba*. Generalized Additive Models of the density of postlarval krill. Effect of additive smoothing functions of (**A**) proportional ice coverage during SUIT hauls, and (**B**) mixed layer depth in summer (2007/2008), (**C**) salinity in the 0–200 m depth layer in autumn (2004), and (**D**) temperature in the mixed layer in winter (2006) on the fitted density of postlarval krill. Dashed lines show 95% confidence intervals of smoothers.

The density of postlarval Antarctic krill in the surface layer was significantly related to different combinations of environmental variables in each sampling season. In summer, parametric terms for average ice thickness and attenuation in the mixed layer, combined with smooth functions of proportional ice coverage during SUIT hauls and MLD obtained a very good model fit, explaining 94.6% of the deviance. The modelled krill density was positively affected by both decreasing ice thickness and attenuation in the mixed layer, an approximate proportional ice coverage >12%, and a MLD <12 m or between 20 and 30 m (Figure 5 A, B; Table 3). In autumn, the most parsimonious model related krill density to the parametric term of water temperature in the mixed layer, and a smooth function for salinity in the upper 200 m. Modelled density was highest at a combination of high water temperatures in the mixed layer and low salinities (Figure 5 C; Table 3). In winter, the best model included the parametric predictors ocean depth and MLD, and a smooth function for the water temperature in the mixed layer. The modelled krill density was positively associated with both increasing depth and MLD and water temperatures in the mixed layer between approximately −1.83°C and −1.76°C (Figure 5 D; Table 3).

**Table 3:** *Euphausia superba*.

Sampling season	Overall model statistics		Model terms			
			Environmental variables	Linear estimate	df	p
Summer	AIC	9.6	Ice thickness	−0.2096		<0.01
	Expl. deviance	94.6%	ATC(MLD)	−2.2845		<0.01
			s(Ice coverage)		3.0	<0.01
			s(MLD)		3.0	<0.01
Autumn	AIC	35.7	Temperature (MLD)	2.0715		<0.01
	Expl. deviance	70.8%	s(salinity 0–200 m)		2.2	0.03
Winter	AIC	59.2	Depth	0.0007		0.01
	Expl. deviance	72.2%	MLDs(temperature (MLD))	0.0261	2.9	0.010.01

Parameters for optimal models relating the density of postlarval krill to environmental variables in summer (2007/2008), autumn (2004) and winter (2006). ATC: attenuation; df: estimated degrees of freedom of smoother; MLD: mixed layer depth; s: Cubic Splines smoother.

#### Comparison with *Thysanoessa macrura*

**Figure 6:**
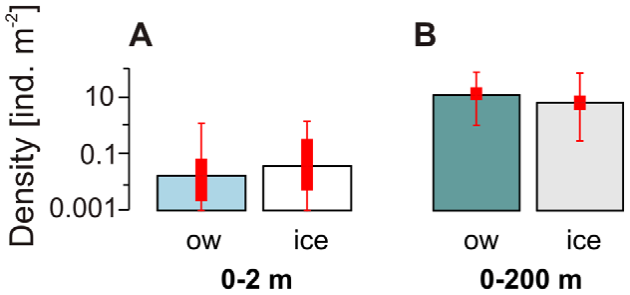
Under-ice versus open water comparison of geometric mean densities of postlarval *Thysanoessa macrura* in summer (2007/2008). (**A**) *Thysanoessa macrura* from the 0–2 m layer, and (**B**) from the 0–200 m layer. Error bars denote value ranges. Bold red bars indicate 25% to 75% percentile ranges. ice = under-ice SUIT hauls; ow = open water SUIT hauls.

Only in summer (2007/2008), postlarval *Thysanoessa macrura* were caught in the 0–2 m surface layer. Occurring at 16 of the 18 quantitative hauls, average surface layer density was below that of Antarctic krill. (0.24 ind. m^−2^), although they locally reached higher densities (Table 4). Overall, the density of *T. macrura* was slightly higher under ice than in open waters. Unlike Antarctic krill, however, the density of *T. macrura* was not significantly related to the presence of sea ice (Figure 6 A; Table 4). In contrast to Antarctic krill, they were only abundant at night and almost not caught at day. The length of postlarval *T. macrura* caught in summer ranged between 8 and 30 mm. They exhibited a unimodal overall size distribution, peaking at 16 mm.

**Table 4:** *Thysanoessa macrura*.

	Summer			Autumn	Winter
	ow	ice	total	total	total (ice)
*n*	7	11	18	16	19
Average	0.18	0.28	0.24	0	0
Geometric mean	0.02	0.04	0.03	0	0
Min	0	0	0	0	0
Max	1.14	1.35	1.35	0	0

Density of postlarval krill [ind. m^−2^] at the open surface and under ice (0–2) m in summer (2007/2008), autumn (2004) and winter (2006). Mean values significantly different from each other (ANOVA *p*<0.05) were printed in bold. ice = under-ice SUIT hauls; ow = open water SUIT hauls; *n* = number of samples.

In the 0–200 m layer, postlarval *T. macrura* occurred in all three seasons. During summer, they were on the average tenfold more abundant in the 0–200 m layer than at the surface (Figure 6 A, B). The density of *T. macrura* was not related to the presence of sea ice (ANOVA: *p*>0.1). In autumn 2004 and winter 2006, very low average densities (<1.0 ind. m^−2^) were recorded in the 0–200 m stratum compared to summer values. In summer, the overall size distributions of the 0–2 m surface and 0–200 m stratum were not significantly different from each other (Kolmogorov-Smirnov test, p>0.05).

## Discussion

### 1. Association of Antarctic krill with sea ice

The present study is the first multi-seasonal investigation of the distribution of Antarctic krill in the top 2 m layer of the ocean, using a micronekton net capable of sampling under closed pack-ice. Covering an area of about 1,000 km in latitudinal, and 200–300 km in longitudinal extent, our results show that significant parts of the Antarctic krill population in the Lazarev Sea can be found in the ice-water interface layer almost year-round, both near the ice edge and hundreds of kilometres deep in the pack-ice (Figure 1). This contrasts with earlier findings suggesting that krill aggregations under ice occur in a narrow band in the marginal ice zone [20].

The potential importance of sea ice habitats for the recruitment and survival of Antarctic krill was highlighted for the south-west Atlantic sector of the Southern Ocean, where populations have been declining in parallel with a decrease in the duration and extent of the winter sea ice coverage during the last 3 decades of the 20^th^ century [16]. This decline is presumably linked to decreasing recruitment success caused by loss of sea ice habitats [46], as larval Antarctic krill are assumed to depend on ice algae to survive their first winter [26], [27], [28]. Our results emphasize the significance of sea ice as an almost year-round key habitat for postlarval Antarctic krill. This supports the notion that postlarval Antarctic krill can sometimes also rely significantly on ice algae as a food source, as well as other organisms thriving in and under the ice [8], [29], [47]. There were, however, significant seasonal differences in the association of Antarctic krill with sea ice, as well as in overall density, size composition, and diel variability. These differences need to be considered in the light of the behavioural plasticity and the life strategy of Antarctic krill.

#### Seasonal patterns of ice association

In summer, high densities of Antarctic krill in the 0–2 m surface layer were significantly associated with the under-ice habitat, rather than the open surface layer (Figure 4 A; Table 1). Antarctic krill's smaller, but often equally abundant sibling species *Thysanoessa macrura* is more omnivorous, and not associated with sea ice [1], [3], [48], [49]. As expected from a species not associated with sea ice, surface layer densities of *T. macrura* were similar under ice and in open water (Figure 6 A), supporting the notion that the observed patterns in Antarctic krill are related to the species' association with the under-ice habitat, rather than to differences in the catch efficiency of SUIT incurred by sea ice. In our model, increasing ice coverage and a shallow mixed layer were positively related to Antarctic krill density in the 0–2 m layer, suggesting that the density of Antarctic krill was highest under melting pack-ice, where a meltwater lens stabilized a shallow mixed layer (Figure 5 A, B; Table 3). These conditions enhance ice algal and phytoplankton production and thus characterise attractive foraging grounds. Accordingly, at low ice coverage the model predicted high krill densities only when the mixed layer was very shallow, concentrating phytoplankton near the surface.

In ice-covered waters, average and geometric mean Antarctic krill densities in the 0–2 m layer were considerably higher than corresponding depth-integrated densities in the 100-fold deeper 0–200 m depth layer (Figure 4 A, B). Many net-based abundance estimates assume that oblique hauls with micronekton trawls, as conducted in our study, sample Antarctic krill quantitatively from the surface down to the maximum sampling depth (e.g. Atkinson et al. 2009 [5]). This assumption, however, implies that depth-integrated overall densities derived from the 0–200 m RMT sampling should be equal or higher than SUIT-based estimates from the 0–2 m surface layer. The much higher arithmetic and geometric mean summer densities in the 0–2 m compared to the 0–200 m water layer in ice-covered waters thus indicate that the ice-water interface layer was probably under-represented in RMT catches. One very likely explanation for this discrepancy is that the RMT sampled in the wake of the ship, where the ice was broken and the upper 10–15 meter of the water column were stirred up by the ship's propellers, whereas the SUIT sampled sideways of the ship's wake under comparatively undisturbed ice (see supporting information S1). The effect of this difference can be expected to be less pronounced when animals are not concentrated at the surface. Accordingly, in open water Antarctic krill were less abundant in the 0–2 m surface layer than in the integrated 0–200 m depth layer, indicating that krill were dispersed over a wider depth range (Figure 4 A, B). In agreement with these considerations, the non-ice-associated *T. macrura* was considerably more abundant in the 0–200 m depth layer compared to the 0–2 m surface layer, irrespective of the presence of sea ice. The comparison of two differently sampled depth layers indicates that a significant proportion of the Antarctic krill population resided immediately under the ice, where it was out of reach of conventional pelagic trawls. Due to an under-representation of the ice-water interface layer, patterns of ice-association evident in the surface layer were not reflected by standardised 0–200 m sampling. Quantitative comparisons between the two depth layers expressing densities as a proportion of the total population, however, should be considered with caution, because the extent to which the two nets sample overlapping depth ranges, as well as differing effects of towing speed, ice properties, and other factors on their catch efficiency are not accurately known.

Even when this caveat is taken into account, our results indicate that Antarctic krill were concentrated in the under-ice habitat both in the horizontal and the vertical dimension during summer. The concentration of Antarctic krill under sea ice observed in the Lazarev Sea during summer suggests that they rely on sea ice biota to a significant extent, as long as ice is present. This pattern, however, may change as soon as rich phytoplankton blooms develop. In more productive shelf-influenced systems, such as the Scotia Sea, Antarctic krill can rely significantly on phytoplankton blooms while sea ice is still present [50]. Phytoplankton is considered the main food of Antarctic krill during summer, but they can also utilise a wide range of other food sources, including copepods, protozoa and detritus [51], [52], [53]. This dietary plasticity is mirrored in their habitat use, which ranges from the open surface and ice-underside down to the deep sea floor [6], [17], [52], [54].

In autumn, postlarval Antarctic krill in the 0–2 m surface layer were more abundant in open water than under ice (Table 1). In this season, the sea ice at the three stations sampled in the ice-covered part of the survey area was young and unlikely to host a sufficiently attractive microbial community for postlarval krill. The low attractiveness of the young sea ice as a foraging ground was reflected in a dispersal of postlarval Antarctic krill over a wider depth range, apparent from the almost consistently higher densities in the 0–200 m stratum (Figure 4 C, D). In the virtual absence of sufficiently attractive pack-ice, the distribution of postlarval Antarctic krill in the 0–2 m surface layer reflected broad-scale hydrographical patterns in autumn (Figure 5 C, Table 3). In contrast, larval Antarctic krill were significantly more abundant under the young autumn sea ice than in open water. Larval Antarctic krill are unable to actively move into more productive waters, and cannot survive starvation during periods of low food availability [28]. Hence, even in young autumn ice, they apparently foraged on sea ice biota, supporting the hypothesis that the distribution patterns of adult and larval Antarctic krill differ most when food availability is low [31].

In winter, by far the highest local densities of Antarctic krill were encountered under the sea ice (Table 1, Figure 1). A comparison between the ice-covered and the open water surface layer was not possible in winter 2006, because the entire survey area was covered by dense pack-ice. Probably due to low variability in the sea ice parameters measured, hydrographical parameters rather than sea ice properties were found to be related to krill density (Figure 5 D; Table 3). The repeated encounter of extra-ordinary high densities with values far above maxima found integrated over the 0–200 m depth layer, indicated that Antarctic krill were associated with the under-ice habitat at least at certain times. The mere absence of Antarctic krill from the ice-water interface layer at day versus high densities at night indicated that the association with the underside of sea ice may be limited to the dark hours in winter (Figure 3 B). During summer, almost the opposite observation was made, independently of the presence of sea ice (Figure 3 A). This demonstrated that the observed diel patterns were not related to differences in the catchability of the SUIT incurred by the presence of daylight and/or sea ice. Such a seasonally divergent diel pattern partly reflects the seasonal shift between low amplitudes close to the surface in summer and high diel vertical migration amplitudes in winter proposed by Siegel (2005) [4], and was corroborated by time series measurements of acoustic zooplankton backscatter in the Lazarev Sea [55]. Although the present study was not designed to allow a sound investigation of diel vertical migration patterns, our results from the surface layer demonstrate that the vertical distribution of Antarctic krill includes the surface layer at all seasons, rather than being largely limited to deeper layers in autumn and winter.

Sea ice has been proposed repeatedly to play a crucial role for the overwintering of Antarctic krill [8], [15], [16]. Our study supports this notion with the first large-scale quantitative evidence of postlarval Antarctic krill dwelling under ice in winter. In a bathymetrically similar area off east Antarctica, Antarctic krill were found feeding on sea ice biota during winter, and being in better condition than animals from open water areas [56]. However, other winter investigations could not find any indication of krill aggregations under ice [24], [57]. These investigations, as well as the high variability of under-ice densities observed in our study during winter, support the increasingly accepted perception that krill combine various strategies to survive the winter, such as reduced metabolism, shrinkage and benthic feeding [34], [35], [58], [59], [60]. The role of sea ice biota in winter feeding may be taken by other food sources, where they are available. In productive shelf areas for example, seabed detritus may be a readily accessible food source [52], [58], resulting in an overall deeper distribution of krill during winter [24], [57], [61]. Using a physiological approach to investigate the overwintering strategy of Antarctic krill in the Lazarev Sea, Meyer et al. (2010) [35] demonstrated that adult krill reduce metabolism and rely on lipid deposits during winter, but at the same time need to feed at low rates to meet their energetic demand. Juvenile Antarctic krill may particularly depend on winter feeding, because they have less storage capacity and metabolic plasticity than older animals [60], [62]. With a maximum size mode at 38 mm resulting from distribution mixture analysis using the CMIX software [63], most Antarctic krill in our dataset were not older than 2 years according to the length-at-age data by Siegel (1987) [64] (Figure 2). Based on the dimensions of the net, the towing speed, and the similarity in euphausiid species composition and size distribution, it can be assumed that the size selectivity of the SUIT did not differ significantly from the well-established RMT (Table 2, supporting information S1). Our results thus indicate that feeding under ice may be important at least for larval and 1–2 year-old postlarval krill in the Lazarev Sea during winter. Populations dominated by older animals, however, may show a different behaviour.

### 2. Implications for Antarctic sea ice ecosystems

In the Lazarev Sea, Antarctic krill seem to concentrate in the ice-water interface layer whenever sea ice is present and biologically mature enough to sustain sufficient resources for grazing. This emphasises the high ecological relevance of the sea ice habitat for Antarctic oceanic ecosystems. Information on the seasonality, spatial distribution and quantitative importance of the association of Antarctic krill with the sea ice habitat has so far been very limited. Investigations using pelagic nets reported both a positive and a negative association of Antarctic krill with ice-covered waters [3], [7], [22], [25]. The ecological importance of the ice-water interface layer, however, may have been considerably underestimated in the past due to limitations to sample this habitat appropriately by pelagic nets and echosounders [37]. An under-estimate by pelagic sampling in the past may have caused ice-covered areas to appear poorer in biological resources than they are in reality. Food demand of the top predator community has been shown to persist or even increase hundreds of kilometres deep into the pack ice, although pelagic primary production there indicated low availability of resources [65]. This pattern was also observed during the present study, supporting the hypothesis that the surface layer, and especially the ice-water interface, might play a crucial role in sustaining the top predator populations of the Antarctic seasonal sea ice zone [65], [66], [67]. The pronounced presence of Antarctic krill under the ice highlights its potential as an energy transmitter between the production of ice algae and the pelagic food web. The trophic relationships among ice algae, Antarctic krill and higher predators, however, are complex. To date, it is unclear to which extent other species are involved in the energy transmission from the ice to the top predators [68], [69], [70], [71]. Clearly, the role of Antarctic krill is shared with a wide spectrum of other species, including fishes, squids, ice-associated copepods, pteropods, chaetognaths and amphipods [37], [72], [73], [74], [75]. In this context, the concentration of Antarctic krill under sea ice should be considered more as an indicator of the ecological potential of the sea ice system than as the sole pathway of energy transmission.

Recently, concern has been expressed that climate change-induced sea ice decline may endanger the sustainability of Antarctic krill populations and associated ecosystems, particularly in the light of an expanding krill fishery [16], [76], [77]. Our results support this notion by demonstrating that the underside of sea ice is a key habitat of Antarctic krill almost year-round in a typical Antarctic oceanic ecosystem. Furthermore, the significance of the ice-water interface layer may have been under-estimated in the past by the pelagic nets and sonars used to estimate the population size of Antarctic krill for management purposes. The present study emphasises the urgent need of a profound understanding of the habitat use of Antarctic krill in the context of its ecological plasticity. Such knowledge is crucial for predictions of the development of krill populations under scenarios of decadal climate oscillations or long-term climate change [46], and an important prerequisite of the ecosystem-based management approach of the Convention on Conversation of Antarctic Marine Living Resources (CCAMLR) [46], [78], [79], [80].

## Supporting Information

Supporting Information S1 Jan Andries van Franeker, Hauke Flores, Michiel van Dorssen: The Surface and Under-Ice Trawl (SUIT). *Detailed description of the novel sampling device used in this study*.
(PDF)
