# Supplementary material for: The Association of Antarctic Krill Euphausia superba with the Under-Ice Habitat
Source: PLoS One. 2012 Feb 23;7(2):e31775. doi: 10.1371/journal.pone.0031775 (PMC3285626; doi:10.1371/journal.pone.0031775)
Supplement: Supporting Information S1 — Jan Andries van Franeker, Hauke Flores, Michiel van Dorssen: The Surface and Under-Ice Trawl (SUIT). Detailed description of the novel sampling device used in this study. (PDF) [file pone.0031775.s001.pdf]

The Surface and Under-Ice Trawl (SUIT)

Jan A. van Franeker, Hauke Flores, Michiel van Dorssen

1. Background. The birds, seals and whales of the Antarctic seasonal sea ice zone ultimately depend to a large extent on primary production from algae growing in the pack-ice [1]. However, how biomass and energy assimilated within the sea ice are transferred into the pelagic food web that connects producers (ice algae) and final consumers (birds and mammals), is not well understood. Animals dwelling in the ice-water interface can naturally be assumed to form an important trophic link between the ice and the pelagic food web. However, this harsh environment is difficult to access with conventional sampling methods. As a first approach to quantitatively collect animals dwelling close under the ice canopy, a new sampling gear was developed, the Surface and Under Ice Trawl (SUIT). SUIT systems were successfully deployed on four cruises with the icebreakers *Polarstern* and *Aurora Australis* so far [2,3].

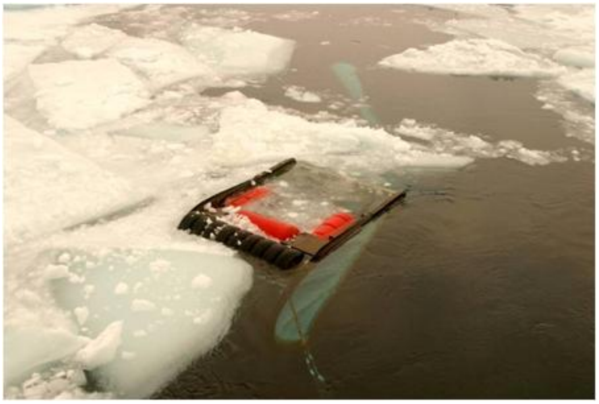

▲ **Figure S1-1.** SUIT in ice during deployment, Lazarev Sea 2006.

2. Construction. The frame consists of 3-inch pipes enclosing an opening of approximately 2*2 m. The front pipes are rounded like the gliding bars of a sledge to allow smooth gliding under ice (Figure S1-1. S1-2). Gliding is further facilitated by a closed row of wheels above the frontal opening. At the rear side of the frame, oblique slide bars conduct big ice chunks out of the frame through the open top. Floaters attached at the top ensure the upper edge of the SUIT to be in contact with the underside of the ice or at surface level in open water, respectively. The starboard side plane of the frame is filled out with 7 mm half-mesh shrimp nets. Canvas fitted into the starboard side plane enhances sideward shearing (Figure S1-3).

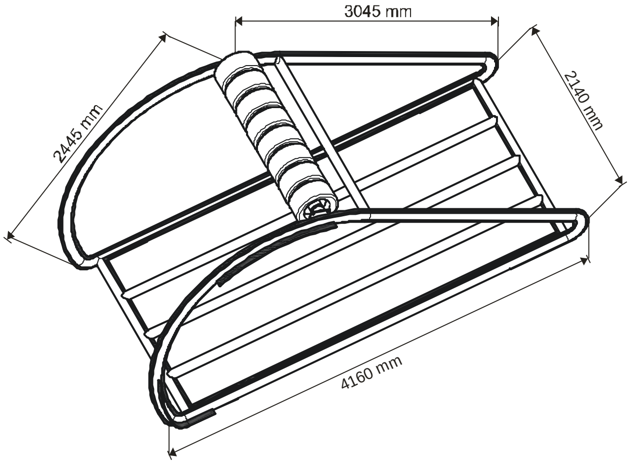

▲ **Figure S1-2.** Technical drawing with major dimensions of the SUIT frame used with RV *Polarstern* in austral summer 2007 / 2008.

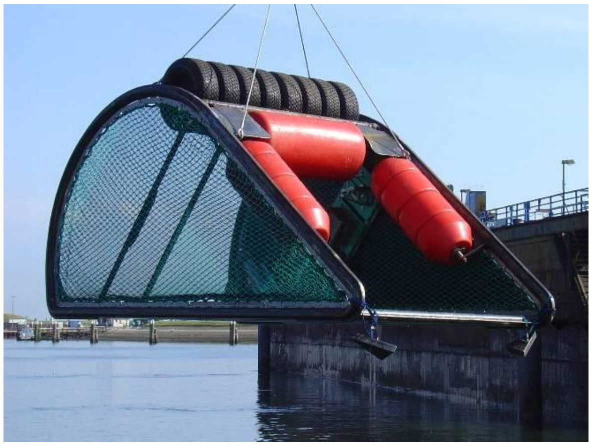

▲ **Figure S1-3.** SUIT frame with floaters and side nets during a test deployment in the harbour of Texel (NL).

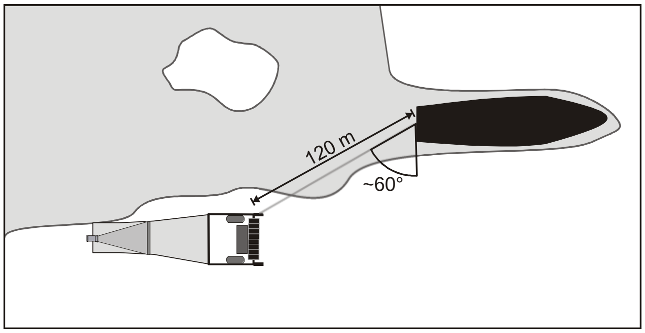

▲ **Figure S1-4.** Sketch of shearing SUIT in bird-eye view.

The bridle is attached only to the portside of the SUIT frame, causing the net to shear out to the starboard side of the ship (Figure S1-4). To avoid interference of the towing cable with ice floes, a 900 kg weight hung on the cable ensures it to stay several meters under water directly behind the vessel (Figure S1-5, S1-6). The position of the weight on the cable is adjustable. In open water, SUIT can be deployed without the weight.

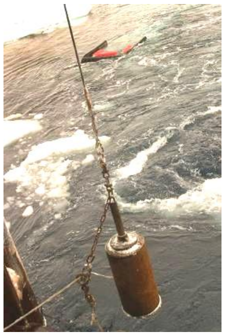

▲ **Figure S1-5.** Deployment of the weight on the towing cable.

▼ **Figure S1-6.** Side view sketch of SUIT with cable and weight.

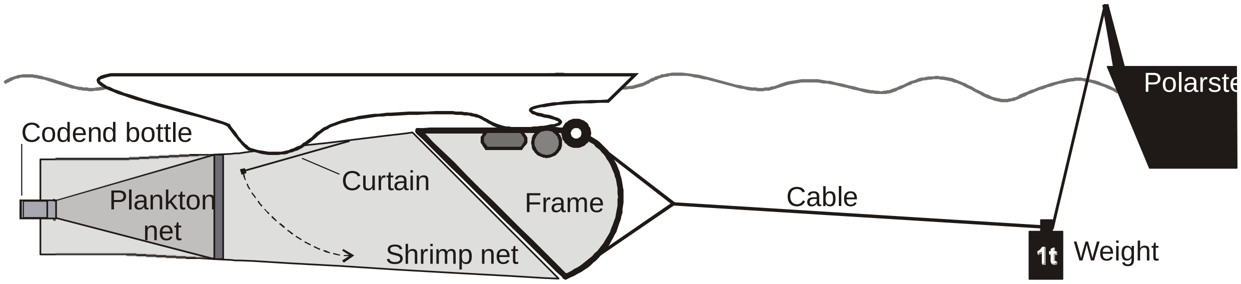

A 7 mm half-mesh shrimp net of approximately 15 m length was used during standard deployments. In the frontal part, a net curtain with a lead line at the bottom closes the net at slow towing speed, impeding animals caught to escape from the net. The rear 3 meters of the net are lined with 0.3 mm plankton gauze connected to the shrimp net with zippers (Figure S1-6). The catch is collected in a 70 cm long codend bottle attached to the end of the plankton net. The approximately 20 cm inner diameter of this bottle creates a still water space during the trawl, protecting the catch from mechanical disruption (Figure S1-7).

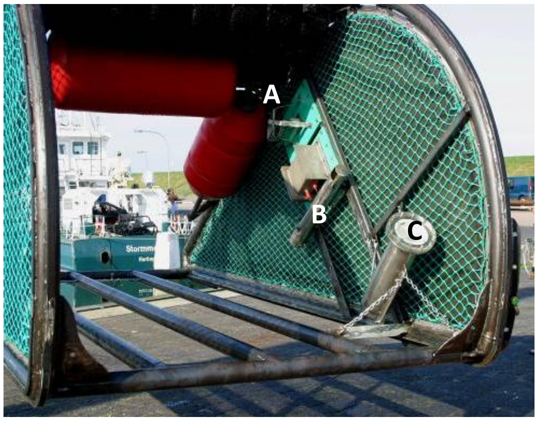

Rails in the SUIT frame allow the attachment of various additional devices. To date, a strobe light, a video system and an acoustic Doppler current profiler (ADCP) have been used with SUIT (Figure S1-8). The latter device served as a standard tool to estimate the amount of water passing through the net.

▶ **Figure S1-7.** Codend bottle attached to plankton net.

◀ **Figure S1-8.** View from the front into the net mouth of the SUIT frame. A camera cage (**A**), an ADCP housing (**B**) and a strobe light (**C**) are mounted at the portside.

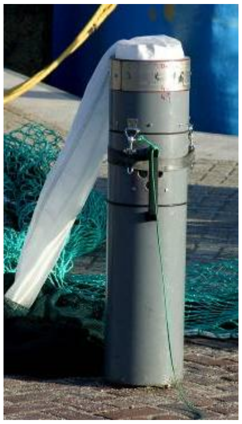

3. Towing performance. In winter 2006 and summer 2007 / 2008, an ADCP was installed in the SUIT frame to allow a detailed analysis of the water flow into the net. The ADCP is able to measure current speed continuously at three different positions across the net opening (Figure S1-9). By standard, the central measurement cell was used to estimate inward directed current velocity (Figure S1-10).

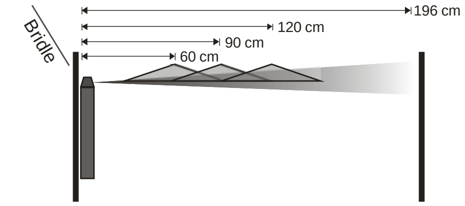

▲ **Figure S1-9.** Position of ADCP measurement cells viewed from top. Triangles show overlapping ranges.

During deployment the towing cable length is increasing, causing SUIT to move considerably slower than the ship. During the phase of steady trawling, fluctuations in shearing slow down the average current velocity inside the frame to about 90 % of the ship's speed in water. Wave action causes moderate oscillations in current speed. During retrieval, the towing cable is taken in, causing SUIT to move faster than the decelerating ship as long as shearing continues (Figure S1-11). Using real-time current speed measurements directly in the net opening as a basis for quantitative calculations inherently accounts for the difference between the water distance sampled compared to the distance covered by the ship.

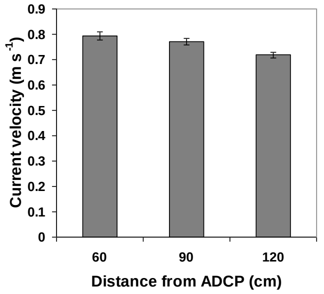

▲ **Figure S1-10.** Example of mean (± s.e.) current velocities in SUIT mouth.

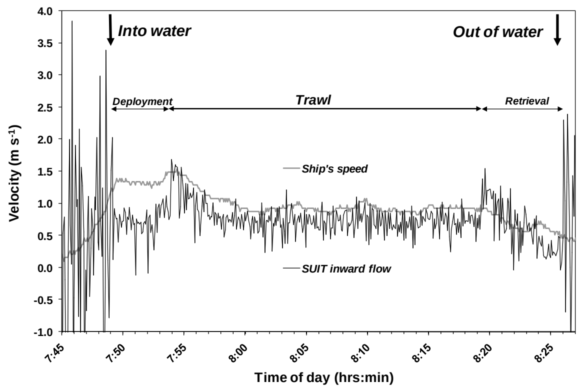

▲ **Figure S1-11.** Current velocity in the SUIT mouth during an open water haul. The ADCP cannot measure outside water.

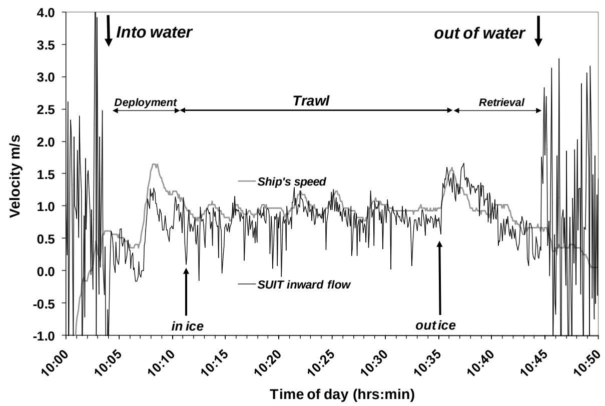

▲ **Figure S1-12.** Current velocity in the SUIT mouth during a haul under sea ice.

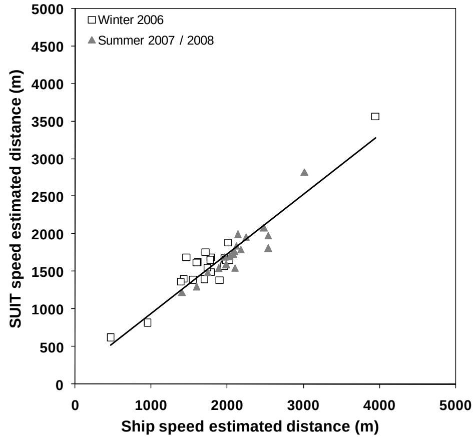

During ice-breaking the ship's speed is more variable than in open water because it needs permanent adjustment to maintain the standard range of trawling speed (1.5 – 2.0 knots = 0.8 – 1.1 m s^{-1}). Impacts of floe edges and under-ice topography cause sudden extreme but short-lasting negative oscillations of inward directed water current (Figure S1-12).

The ADCP flow measurements were used to calculate the distance of water filtered by SUIT. The reliable relationship with the sampled distance estimated from the ship's speed in water allowed the reconstruction of the distance of water sampled by SUIT for autumn 2004, when no ADCP was used (Figure S1-13).

◄ **Figure S1-13.** Relationship of the distance passed through water estimated from the ship's speed in water with distance estimated from current speed in the SUIT.

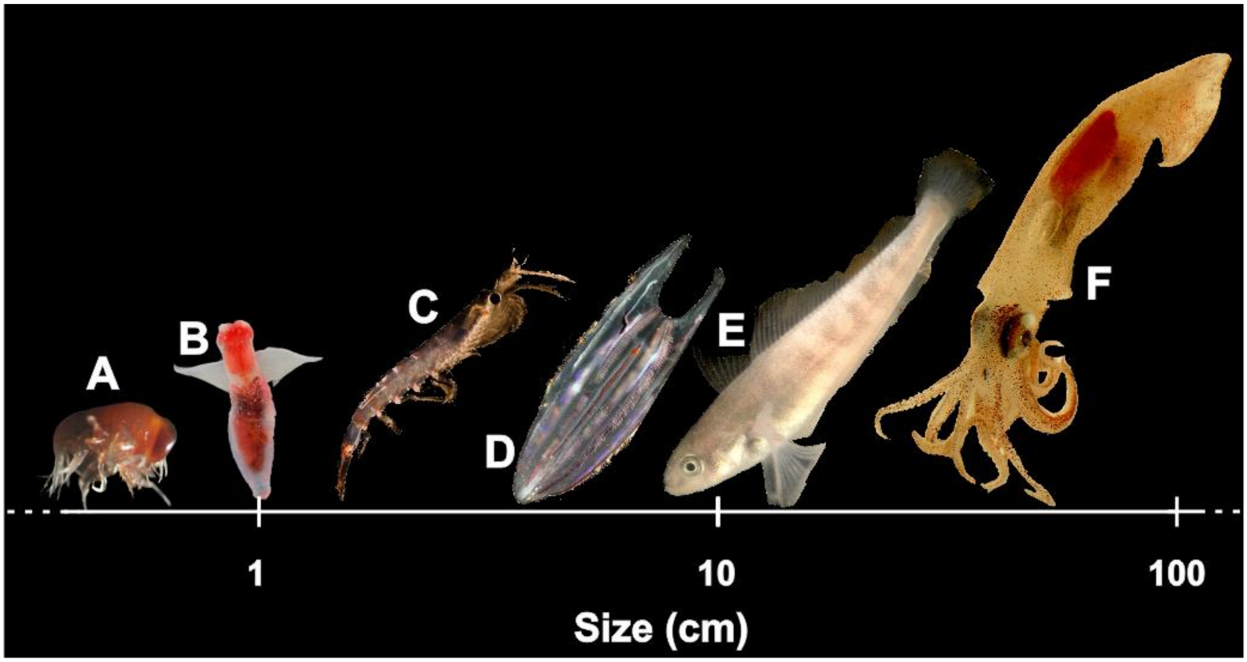

▲ **Figure S1-14.** Examples of macrofauna caught with SUIT. **A:** amphipod *Hyperiella dilatata*; **B:** sea angel *Clione limacina antarctica*; **C** Antarctic krill *Euphausia superba*; **D:** comb jellyfish *Callianira antarctica*; **E:** threadfin pithead *Aethotaxis mitopteryx*; **F:** squid *Slosarcszykovia circumantarctica*.

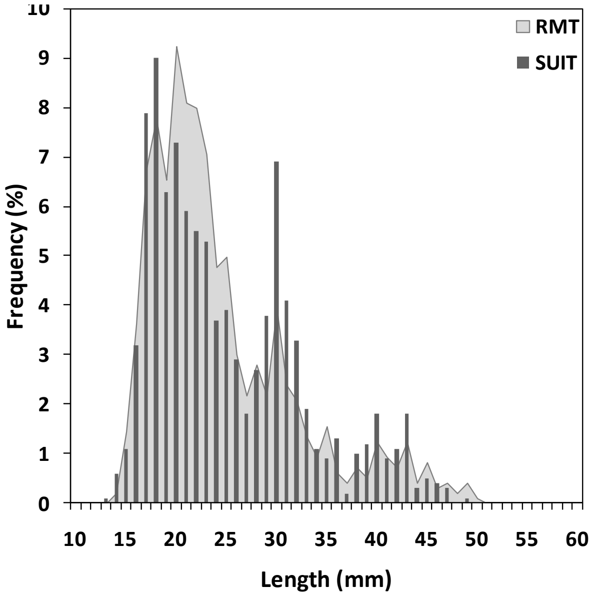

▲ **Figure S1-15.** Length-frequency distributions of Antarctic krill *Euphausia superba* caught with SUIT and RMT at identical locations in the Lazarev Sea in austral summer 2007 / 2008.

4. Catch composition. A wide spectrum of plankton and nekton species was caught with SUIT ranging in size from ostracods (< 1 mm) to adult squid (420 mm) (Figure S1-14; [2]). The biggest fish caught with SUIT was a snake pipefish *Entelurus aequoreus* (390 mm) collected during a test haul in the Dutch Wadden Sea [4]. The most frequently caught macrofauna species was Antarctic krill *Euphausia superba*.

There was no significant difference between the size compositions of krill caught by SUIT and krill caught by the well-established rectangular midwater trawl (RMT) in three sampling seasons (Figure S1-15). This similarity indicates that there was no significant difference in the size selectivity of the two nets.

SUIT catches from summer 2007 / 2008 yielded higher densities at day than at night, indicating that krill could not avoid the net even at optimum visibility (S1-16). Although direct evidence is still lacking, these results strongly support the assumption that SUIT is able to sample Antarctic krill and other micronekton species quantitatively.

Uncertainty, however, remains for the catch efficiency for true nekton (adult fish and squid). For example, the density of the lanternfish *Electrona antarctica* was much higher in RMT than in SUIT catches in autumn 2004 [2,5], leaving unclear whether only a small proportion of the population reached the surface layer or most fish could avoid the net. Although more clarity is needed with regard to these limitations, SUIT provides a new opportunity of direct quantitative sampling in the ice-water interface layer and at the open surface with the same gear at least for zooplankton and micronekton.

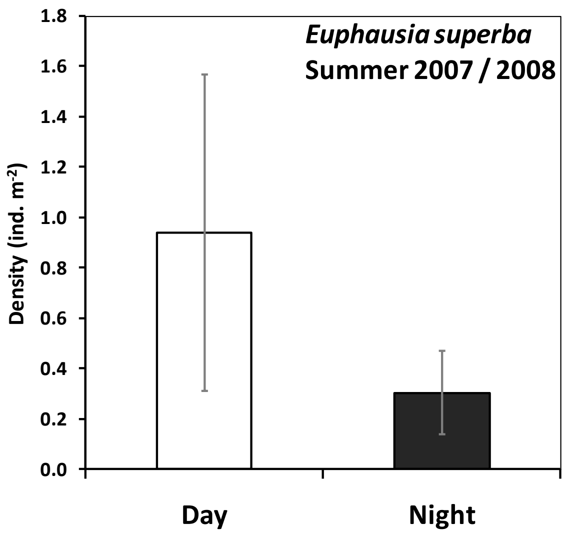

▲ **Figure S1-16.** Mean (± s.e.) density of day / night comparisons of postlarval Antarctic krill at three Locations sampled by SUIT in the Lazarev sea, austral summer 2007 / 2008.

SUIT was designed by Jan Andries van Franeker and built by M van Dorssen Metaalbewerking (Texel, The Netherlands).

Detailed information about SUIT is available on request from J.A. van Franeker, IMARES.

Acknowledgements

The following people and companies supported Wageningen IMARES and van Dorssen Metaalbewerking in the development and application of SUIT:

A. Meijboom, R. Fijn, B. Fey, J. Zegers, U. Bathmann, A.P. van de Putte, *Polarstern* crews of ANT XXI/4, ANT XXIII/6 and ANT XXIV/2 (particularly U. Grundmann, B. Clasen, D. Beth, K. Hoppe), V. Siegel , crew of RV *Navicula*, D. de Haan, J. Betsema, A. Boon, R. Wilzewski, the Alfred Wegener Institute for Polar and Marine Research (AWI), C.I.V. Texel b.v., Zeilmakerij Texel, Schat Harding GmbH, Qmetrix b.v.. SUIT development and application was funded by the Netherlands AntArctic Programme (NAAP Project nr. 851.20.011) and the Dutch Ministry for Agriculture, Nature and Food Quality (LNV) through its Statuary Research Tasks Programme (WOT project 04-003).

References

1. Brierley AS, Thomas DN (2002) Ecology of Southern Ocean pack ice. Advances in Marine Biology 43: 171-276.

2. Flores H, van Franeker JA, Cisewski B, Leach H, Van de Putte A, et al. (2011) Macrofauna under sea ice and in the open surface layer of the Lazarev Sea, Southern Ocean. Deep-Sea Research Part II: Topical Studies in Oceanography: doi:10.1016/j.dsr2.2011.01.010

3. O'Brien C, Virtue P, Kawaguchi S, Nichols PD (2011) Aspects of krill growth and condition during late winter-early spring off East Antarctica (110-130°E). Deep-Sea Research Part II: Topical Studies in Oceanography 58: 1211-1221.

4. van Franeker JA, Flores H (2007) Zuidpool en zeenaalden? Nederlandse Zeevogelgroep Nieuwsbrief 8: 4.

5. Flores H, Van de Putte A, Siegel V, Pakhomov E, Van Franeker J, et al. (2008) Distribution, abundance and ecological relevance of pelagic fishes in the Lazarev Sea, Southern Ocean. Marine Ecology Progress Series 367: 271-282.
